# Supplementary material for: Evaluating E. coli genome‐scale metabolic model accuracy with high‐throughput mutant fitness data
Source: Mol Syst Biol. 2023 Oct 27;19(12):e11566. doi: 10.15252/msb.202311566 (PMC10698504; doi:10.15252/msb.202311566)
Supplement: Supplementary file 1 — Appendix [file MSB-19-e11566-s001.pdf]

Appendix

Table of Contents

Appendix Figure S1: Experimental fitness and simulation results..4

Appendix Figure S2: Experimental fitness and post-correction simulation results..........................7

Appendix Figure S3: Machine learning training and cross-validation..8

Appendix Figure S4: SHAP value dependency plots for select flux features....................................9

Appendix Table S1: Vitamin/cofactor biosynthetic pathway mutants' fitness after 5 or 12 generations..10

Appendix Table S2: Presence of vitamin/cofactor in biomass equations of *E. coli* GEMS............11

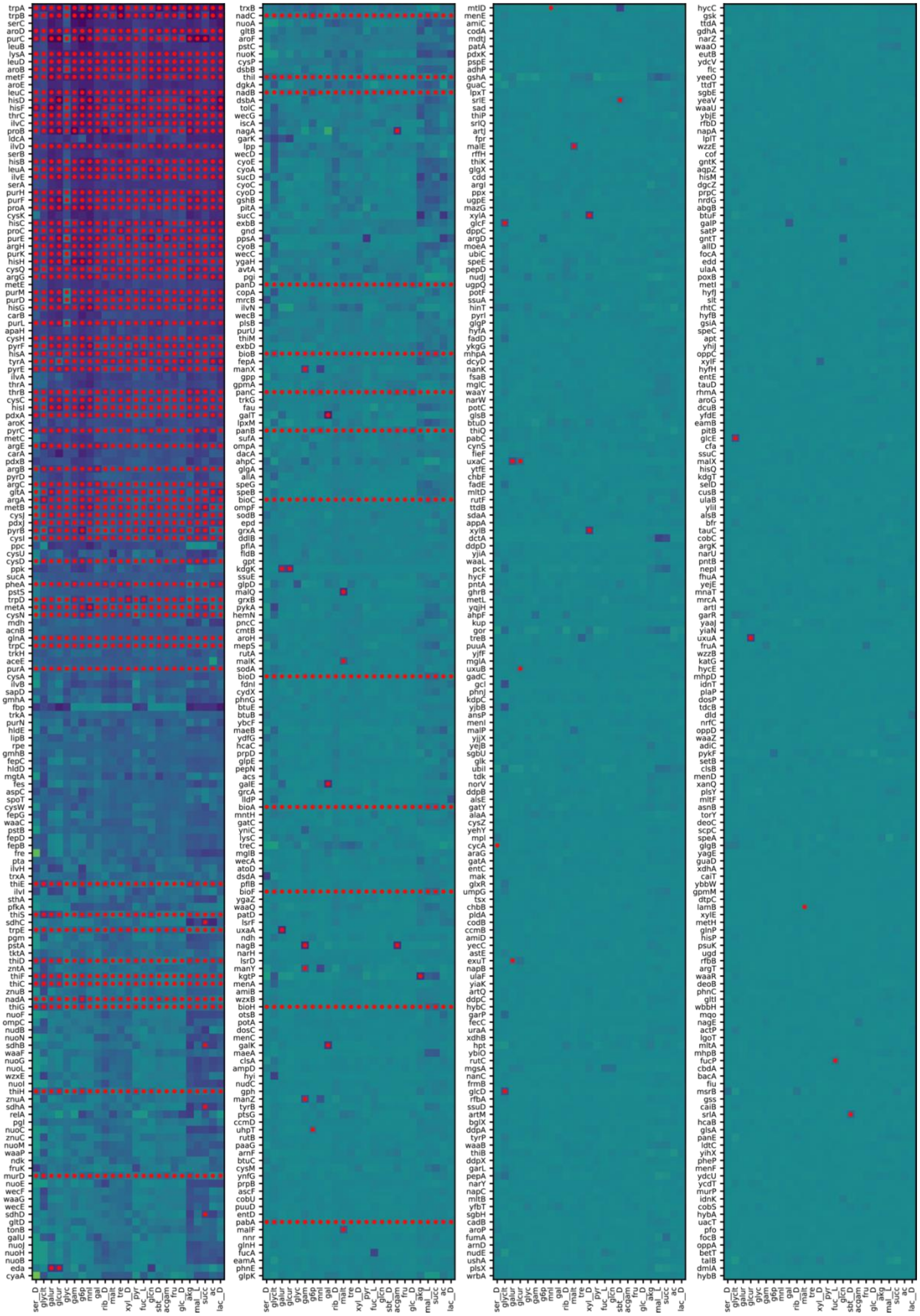

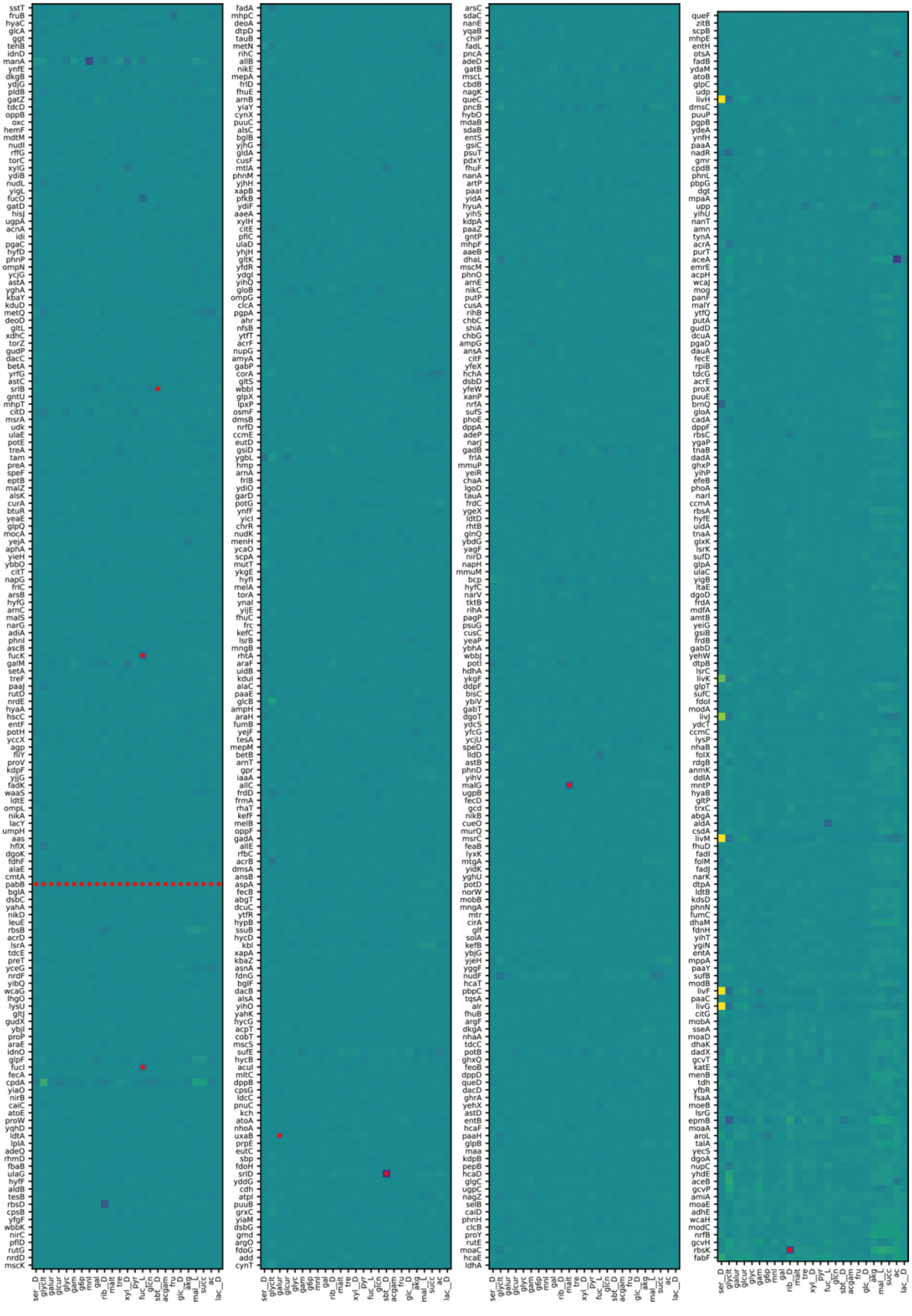

Appendix Figure S1: Experimental fitness and simulation results
The entire data matrix of genes by carbon sources is visualized. Color indicates experimental fitness value (dark blue: low, yellow: high), a red dot indicates simulated no-growth (biomass flux < 0.001).

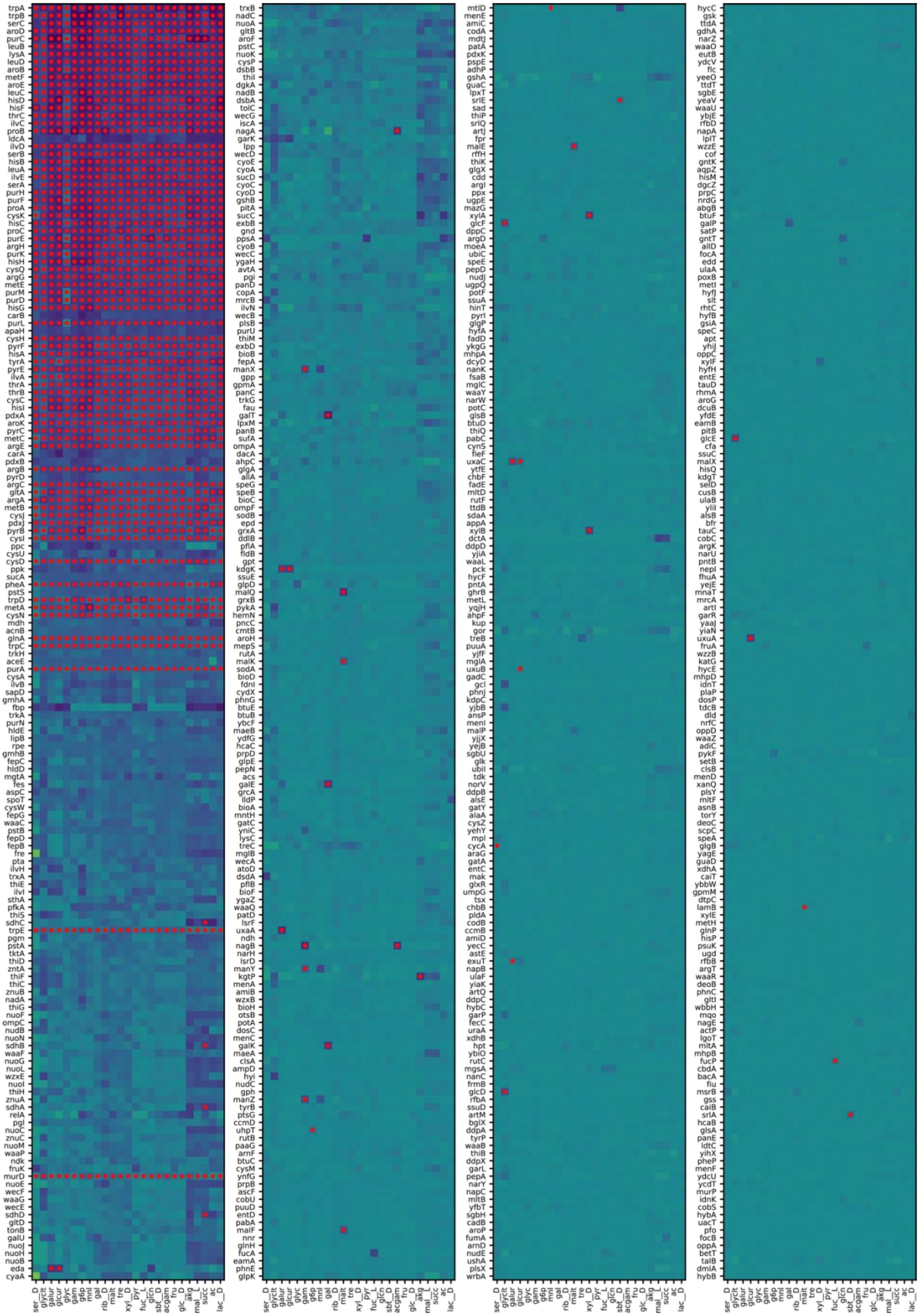

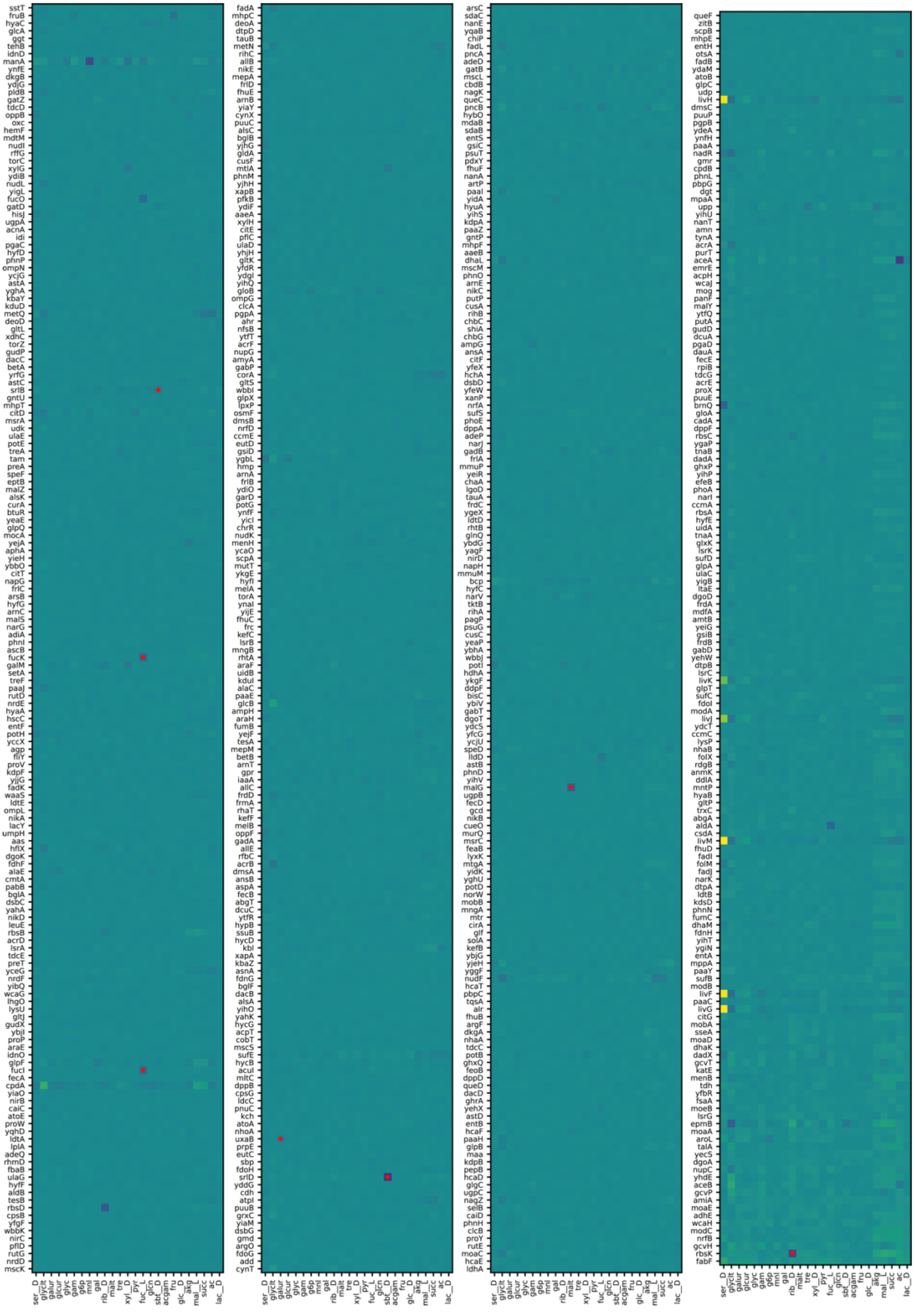
ser_D
glyclt
galur
glcur
glyc
gam
g6p
mnl
gal
rib_D
malt
tre
xyl_D
pyr
fuc_L
glcn
sbt_D
acgam
fru
glc_D
akg
mal_L
succ
ac
lac_D

Appendix Figure S2: Experimental fitness and post-correction simulation results
The entire data matrix of genes by carbon sources is visualized. Color indicates experimental fitness value (dark blue: low, yellow: high), a red dot indicates simulated no-growth after implementing corrections (biomass flux < 0.001).

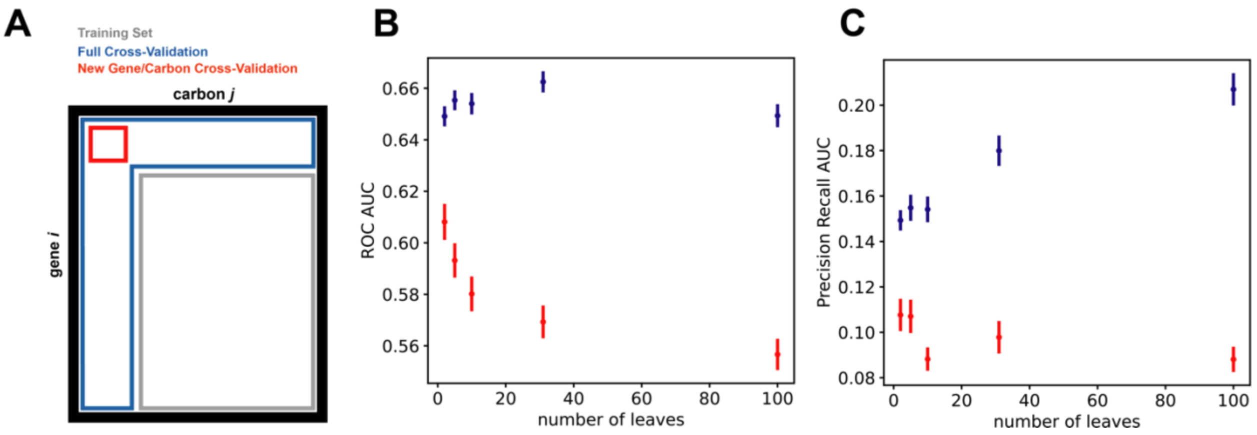

Appendix Figure S3: Machine learning training and cross-validation

A) The schematic for training and test set partitioning is shown. The data is structured as a matrix of genes by carbon sources (with a vector of metabolic fluxes for each element in this matrix serving as the machine learning input features). The test error was calculated in two different ways. First, for all held out experiments in a full cross-validation with 80% of the carbon sources and 80% of the genes included in the training set (training set: grey outline, test set: blue outline). Second, for new gene/carbon sources such that there is no overlap in genes/carbon sources between the training and test set (training set: grey outline, test set: red outline).

B) The area under the receiver operating curve accuracy of the machine learning model for different numbers of leaves (an important parameter to control overfitting) is plotted for 100 random train/test splits. Mean +/- standard error is shown.

C) The area under the precision-recall curve accuracy of the machine learning model for different numbers of leaves is plotted for 100 random train/test splits. Mean +/- standard error is shown.

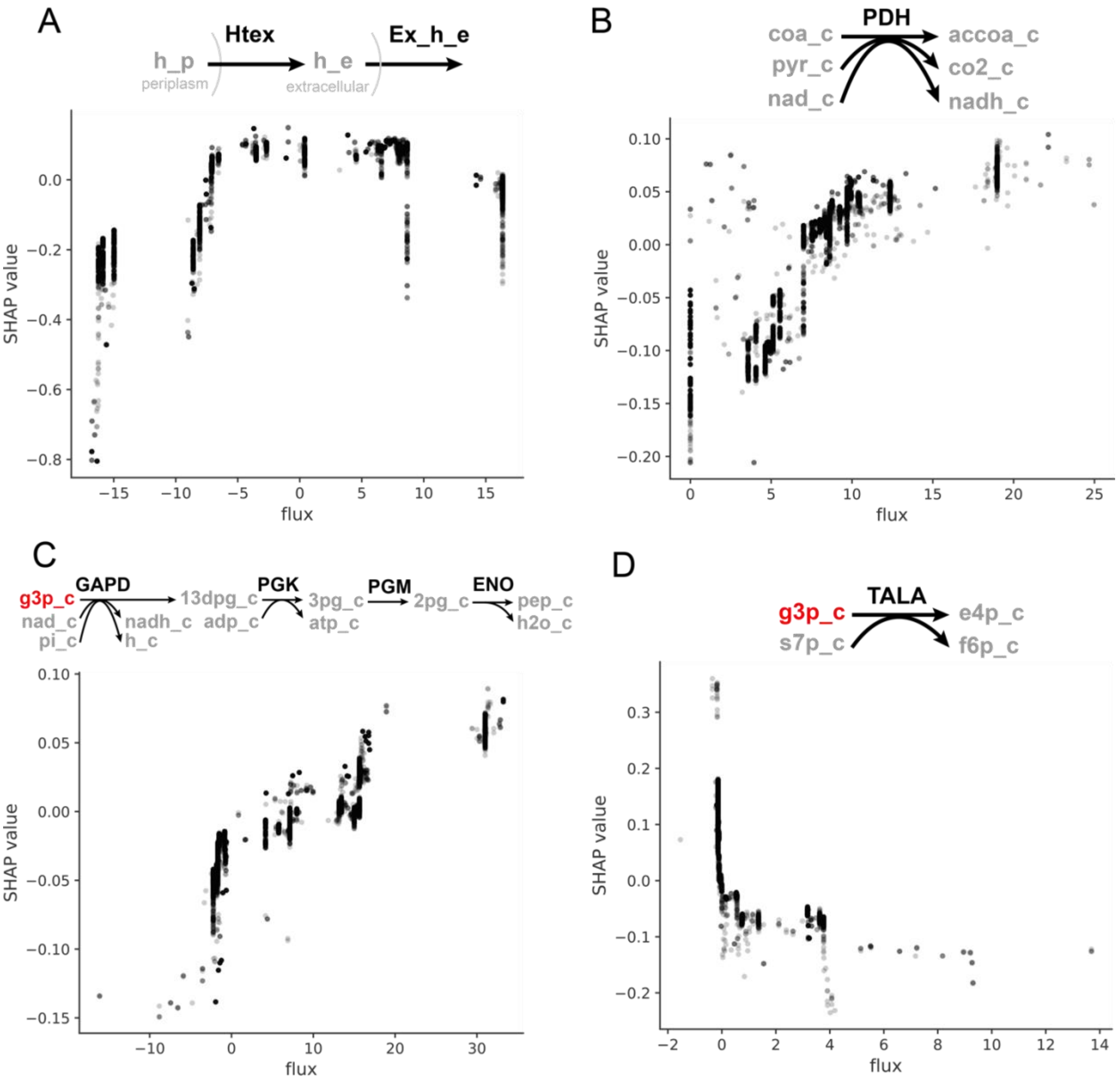

Appendix Figure S4: SHAP value dependency plots for select flux features

A) Hydrogen ion exchange and transport

B) Pyruvate dehydrogenase

C) Lower glycolysis

D) Transaldolase

Appendix Table S1: Vitamin/cofactor biosynthetic pathway mutants' fitness after 5 or 12 generations

The original data published in (Price et al. 2016) is concisely presented here.

Gene ID	Gene Name	Fitness (5 generations, replicate A)	Fitness (5 generations, replicate B)	Fitness (12 generations, replicate A)	Fitness (12 generations, replicate B)
Biotin		fit6A	fit6B	fit12A	fit12B
b0774	bioA	-0.249733271	0.169883592	-1.725674709	-1.562545914
b0775	bioB	-0.332020237	-0.723814699	-2.387709472	-2.073829501
b0776	bioF	0.066472425	-0.058348875	-1.395538493	-1.279986631
b0777	bioC	-0.146592681	-0.298656474	-1.611090426	-1.488403318
b0778	bioD	-0.039585108	-0.399383656	-1.805952805	-1.412528307
b3412	bioH	-0.251522678	-0.232278713	-1.457237518	-1.275979793
R-Pantothenate					
b0133	panC	-1.866259241	-2.066444309	-6.424696572	-6.089171774
b0134	panB	-1.412537088	-1.901542713	-6.152284976	-6.369511582
Thiamin					
b0417	thiL	NA	NA	NA	NA
b4407	thiS	NA	NA	NA	NA
b2103	thiD	-0.547350783	-0.74018164	-7.192968053	-6.240102165
b3990	thiH	-0.754285217	-0.766960492	-6.544794852	-5.812424961
b3991	thiG	-0.823797977	-0.891640748	-6.635548249	-6.305468588
b3992	thiF	-0.752702277	-0.852329707	-7.157378331	-5.933854313
b3993	thiE	-0.599464474	-0.658769675	-7.437931254	-6.255330043
b3994	thiC	-0.746162696	-0.692587548	-6.91073834	-6.486176817
Tetrahydrofolate					
b3360	pabA	-0.215533894	-0.201223886	-1.067309078	-1.012191424
b1812	pabB	-0.544316429	-0.473292477	-1.083868507	-1.132341867
NAD+					
b2574	nadB	-0.767605052	-1.037471671	-4.754961216	-4.85302795
b0109	nadC	-0.934231932	-1.249495358	-4.765742738	-4.738788867
b0750	nadA	-0.978227963	-1.288170421	-6.389177546	-6.063831515

Appendix Table S2: Presence of vitamin/cofactor in biomass equations of *E. coli* GEMS

The presence of each vitamin/cofactor, or its product, as a consumed metabolite in the biomass equation of each *E. coli* GEM is shown (No – not present, Yes – present). BiGG identifiers, as used in the models, are shown here for metabolite names. The presence of each of the conditionally essential genes identified in our analysis is also shown below along with their essentiality in a simulated minimal medium with no vitamins or cofactors added (**X** – not in the model, **U** - removed because of unconditional essentiality, **N** – non-essential, **E** – essential).

**Vitamin/Cofactor**	**Biomass Component**	**iJR904**	**iAF1260**	**iJO1366**	**iML1515**
btn_c	btn_c	No	No	Yes	Yes
pnto__R_c	coa_c	Yes	Yes	Yes	Yes
thm_c	thmpp_c	No	Yes	Yes	Yes
thf_c	thf_c	No	Yes	Yes	Yes
nad_c	nad_c	Yes	Yes	Yes	Yes
	**Biosynthetic Genes**	**iJR904**	**iAF1260**	**iJO1366**	**iML1515**
	bioH	**X**	**X**	**E**	**E**
	bioD	**N**	**N**	**E**	**E**
	bioF	**N**	**N**	**E**	**E**
	bioC	**X**	**X**	**E**	**E**
	bioA	**N**	**N**	**E**	**E**
	bioB	**N**	**N**	**E**	**E**
	panB	**E**	**E**	**E**	**E**
	panC	**E**	**E**	**E**	**E**
	thiI	**N**	**E**	**E**	**E**
	thiF	**N**	**E**	**E**	**E**
	thiH	**N**	**E**	**E**	**E**
	thiD	**N**	**E**	**E**	**E**
	thiG	**N**	**E**	**E**	**E**
	thiS	**N**	**E**	**E**	**E**
	thiC	**N**	**E**	**E**	**E**
	thiE	**N**	**E**	**E**	**E**
	pabB	**U**	**U**	**U**	**E**
	pabA	**U**	**U**	**U**	**E**
	nadB	**E**	**E**	**E**	**E**
	nadC	**E**	**E**	**E**	**E**
	nadA	**E**	**E**	**E**	**E**
