# Supplementary material for: Evaluating E. coli genome‐scale metabolic model accuracy with high‐throughput mutant fitness data
Source: Mol Syst Biol. 2023 Oct 27;19(12):e11566. doi: 10.15252/msb.202311566 (PMC10698504; doi:10.15252/msb.202311566)
Supplement: Supplementary file 2 — Expanded View Figures PDF [file MSB-19-e11566-s003.pdf]

# Expanded View Figures

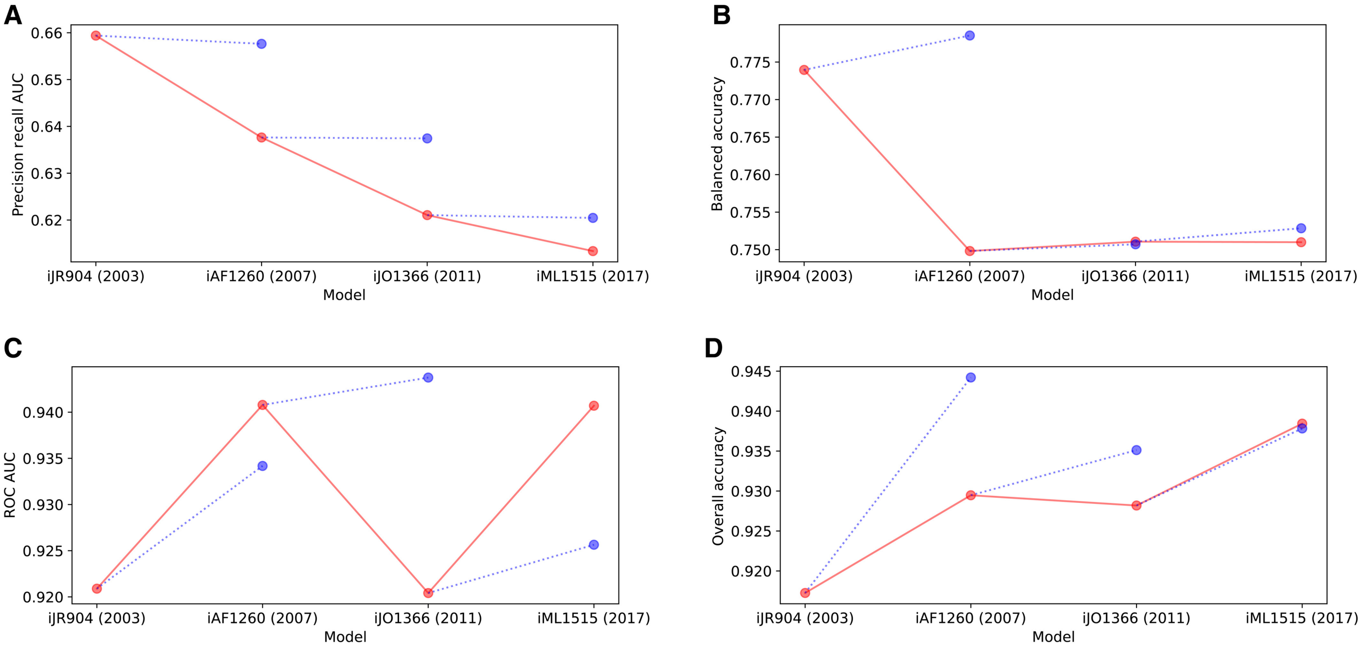


**Figure EV1. Comparison of *E. coli* GEM accuracy for four subsequent versions of the model with alternative metrics and a null model.**

The accuracies of the *E. coli* GEMs are plotted across model version (red, solid line) against a null model (blue, dotted line). The null model quantifies the expected change in model accuracy of each subsequent model assuming that all additional genes are nonfunctional (fitness value of 0, knockout has no effect on simulated growth/no-growth).

A Area under precision-recall curve.
B Balanced accuracy.
C Area under receiver operating characteristic curve.
D Overall accuracy.

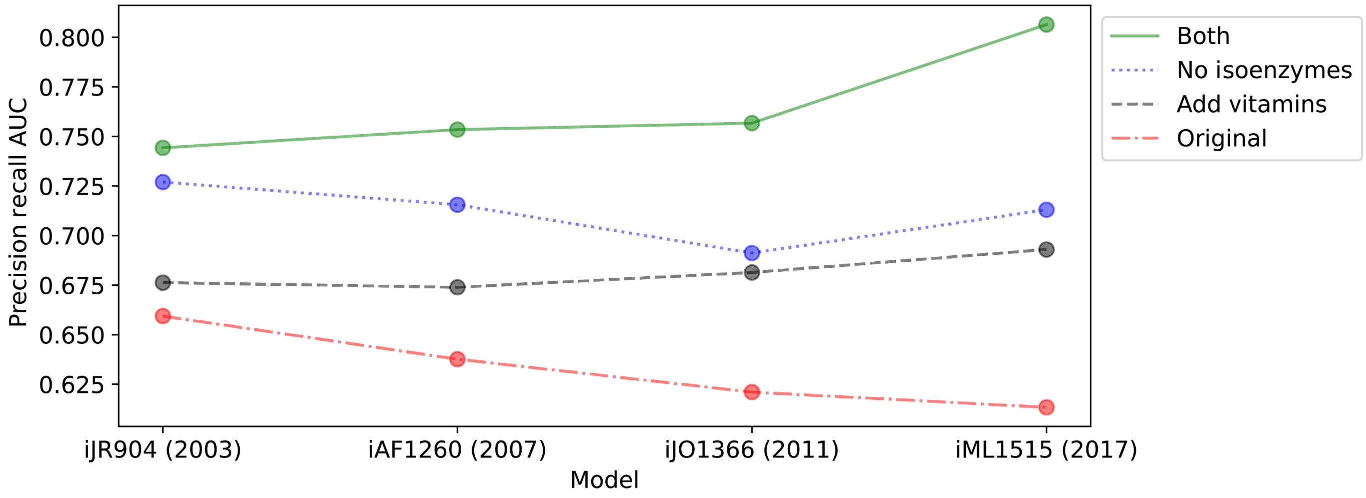


**Figure EV2. Comparison of *E. coli* GEM accuracy for four subsequent versions of the model with corrections.**

Model accuracy, as measured by area under precision–recall curve, is shown for several different corrections to the model analysis approach. The original simulations are shown (red, dash-dot line, same data as shown in Fig 1D). Simulations with vitamins/cofactors added to the model (biotin, R-pantothenate, thiamin, tetrahydrofolate, and $NAD^+$ a were all added through intracellular exchange) are shown (black, dashed line). Quantifications of models' accuracies excluding isoenzymes (any gene with an "or" statement in any associated gene-protein-reaction mapping) are shown (blue, dotted line). Simulations with vitamins/cofactors added and excluding isoenzymes are shown (green, solid line).

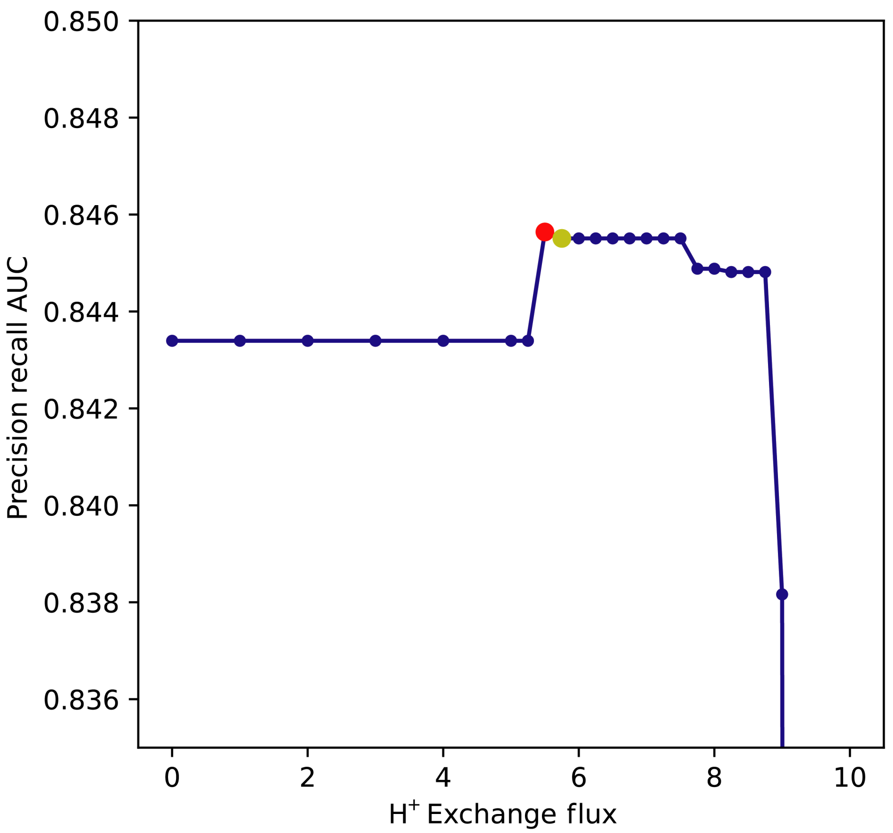


**Figure EV3. Model prediction accuracy with fixed hydrogen ion flux.**

Fixing the bounds of hydrogen ion flux to small positive values (dots show tested values) leads to an optimal value that improves model prediction performance. The first uptick in performance comes from genes in the succinate dehydrogenase complex becoming essential for growth on acetate (red dot, genes and fitness: sdhC −5.682, sdhD −4.306, sdhA −4.303, sdhB −4.25). The second level of improved prediction performance additionally causes genes in the cytochrome complex to become essential for growth on glycolate (yellow dot, genes and fitness: cyoD −2.330, cyoC −3.033, cyoB −2.762, cyoA −2.787). Note that despite the negative fitness of the cyoA-D/glycolate experiments this change to the model leads to a very small decrease in the precision-recall AUC due to the quantitative, threshold-independent nature of the precision–recall AUC calculation. Further increases in the fixed hydrogen ion flux sharply decrease model prediction performance.


© 2023 The Authors
